# Long-range GABAergic projections from the nucleus of the solitary tract

**DOI:** 10.1186/s13041-021-00751-4

**Published:** 2021-02-19

**Authors:** Mei-Yu Shi, Lu-Feng Ding, Yu-Hong Guo, Yu-Xiao Cheng, Guo-Qiang Bi, Pak-Ming Lau

**Affiliations:** 1 CAS Key Laboratory of Brain Function and Disease, and School of Life Sciences, University of Science and Technology of China, Hefei, Anhui China; 2 CAS Center for Excellence in Brain Science and Intelligence Technology, Shanghai, China; 3 CAS Key Laboratory of Brain Connectome and Manipulation, Interdisciplinary Center for Brain Information, The Brain Cognition and Brain Disease Institute, Shenzhen Institutes of Advanced Technology, Chinese Academy of Sciences, Shenzhen, Guangdong China; 4 Shenzhen-Hong Kong Institute of Brain Science-Shenzhen Fundamental Research Institutions, Shenzhen, Guangdong China

**Keywords:** Nucleus of the solitary tract, GAD2 inhibitory neuron, Long-range projection, Paraventricular hypothalamic nucleus, Bed nuclei of the stria terminalis, VISoR

## Abstract

The nucleus of the solitary tract (NTS) plays a crucial role in integrating peripheral information regarding visceral functions. Glutamate decarboxylase 2 (GAD2) inhibitory neurons are abundant in the NTS, and are known to form local and short-range projections within the NTS and nearby hindbrain areas. Here we performed whole-brain mapping of outputs from GAD2 neurons in the NTS using cell-type specific viral labeling together with ultrahigh-speed 3D imaging at 1-μm resolution. In addition to well-known targets of NTS GAD2 neurons including the principle sensory nucleus of the trigeminal (PSV), spinal nucleus of the trigeminal (SPV), and other short-range targets within the hindbrain, the high sensitivity of our system helps reveal previously unknown long-range projections that target forebrain regions, including the bed nuclei of the stria terminalis (BST) involved in stress and fear responses, and the paraventricular hypothalamic nucleus (PVH) involved in energy balance and stress-related neuroendocrine responses. The long-range projections were further verified by retrograde labeling of NTS GAD2 neurons with cholera toxin B (CTB) injections in the BST and PVH, and by Cre-dependent retrograde tracing with rAAV2-retro injections in the two regions of GAD2-Cre mice. Finally, we performed complete morphological reconstruction of several sparsely labeled neurons projecting to the forebrain and midbrain. These results provide new insights about how NTS might participate in physiological and emotional modulation.

The nucleus of the solitary tract (NTS) is an important integrator of peripheral information regarding cardiovascular, respiratory, gastrointestinal and other visceral functions [1–3]. In the NTS, a significant portion of cells are known to be GABAergic neurons, forming an interconnected inhibitory network [4, 5]. Activation of this GABAergic network could cause cardiovascular activation and respiratory inhibition [6, 7]. Early anatomical studies using biocytin labeling suggest that GABAergic neurons in the NTS are interneurons, projecting only locally within the nucleus [8]. Using transgenic mice combined with cell-type viral tracing, more recent work found that GAD2-positive GABAergic neurons in the NTS also send projections to nearby regions, including the medulla and pons [9]. Meanwhile, non-specific anterograde tracing using Phaseolus vulgaris‐leucoagglutinin (PHA-L) has shown that NTS neurons can project to midbrain and forebrain structures [10]. However, it is unclear whether GAD2 neurons in the NTS can form long-range projections, which might have been overlooked in previous studies due to limitations of imaging techniques. In this study, we combined cell type-specific viral tracing with newly-developed high-resolution 3D fluorescence imaging to map the projections of the GABAergic NTS neurons at the scale of the entire brain.

We selectively labeled GAD2 neurons by local injection of AAV-EF1α-DIO-eGFP into the NTS of GAD2-Cre mice (Fig. 1a, a1-a3; Additional file 1: Fig. S1). Brain-wide axonal projections of the eGFP-expressing neurons were examined four weeks after injection using our high-speed 3D imaging system implementing Volumetric Imaging with Synchronized on-the-fly-scan and Readout (VISoR) technology (see “Methods”) [11]. We found that the majority of NTS GAD2 neurons had short-range projections targeting brain regions in the pons and medulla, including the principal sensory nucleus of the trigeminal (PSV), spinal nucleus of the trigeminal (SPV), parvicellular reticular nucleus (PARN) and dorsal column nuclei (DCN) (Fig. 1a, a1, b). These observations are consistent with the descriptions in the Allen Brain Atlas [9].

**Fig. 1:**
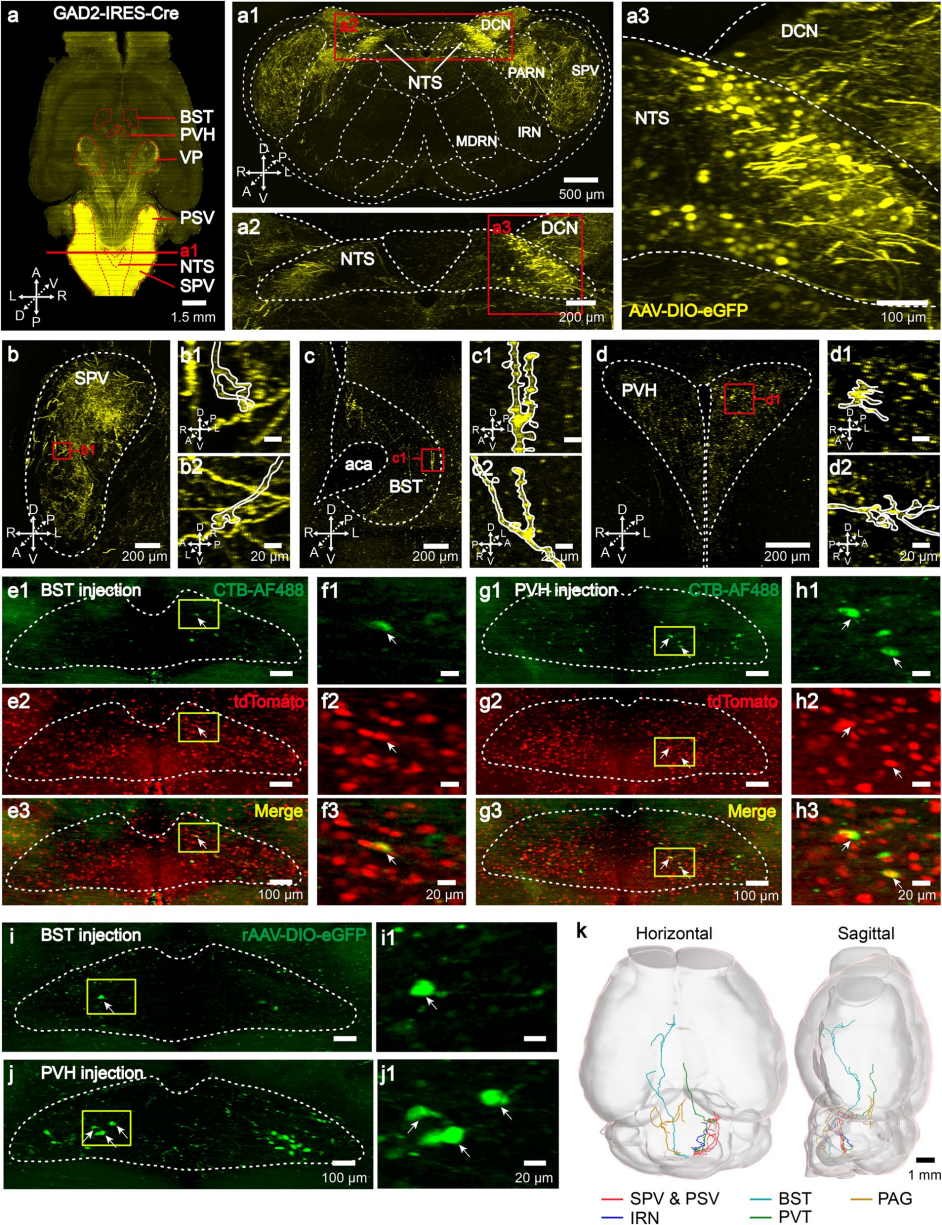
Long-range projections of NTS GAD2 neurons. **a** Horizontal view of the whole-brain projections from NTS GAD2 neurons. Brain regions labeled by dotted lines indicate the AAV injection site in the NTS and terminal-targeted regions in the BST, PVH, VP, PSV and SPV. A, anterior; P, posterior; L, left; R, right; D, dorsal; V, ventral. **a1** Coronal section at the position in (**a**) showed infections of AAV-DIO-eGFP in the NTS, and axonal projections in the nearby regions such as the SPV, PARN, IRN and medullary reticular nucleus (MDRN); boxed regions are magnified to show the details (**a2-a3**). **b**–**d** Maximum-intensity projections of coronal sections showing axonal terminals originating from NTS GAD2 neurons in the SPV (**b**), BST (**c**) and PVH (**d**). Images are maximal projections of 128-μm z-stacks. **b1**, **c1**, **d1** Magnification of axonal boutons in the SPV (**b1**), BST (**c1**) and PVH (**d1**) at the frames indicated in (**b**), (**c**) and (**d**), respectively. **b2**, **c2**, **d2** Sagittal view of the frames indicated in (**b1**, **c1**, **d1**). **e**, **g** Representative images showing expressions of tdTomato fluorescence in GAD2 neurons and CTB-AF488 signal in neurons retrogradely traced from the BST (**e1**–**e3**) and PVH (**g1–g3**). Images are maximal projections of 100-μm z-stacks. **f**, **h** Magnified views of the boxed areas in **e** and **g** showing colocalizations of CTB and tdTomato signals. Arrows indicated GAD2-positve CTB neurons. **i**, **j** Fluorescent somas in the NTS indicated GAD2 neurons projecting to the BST (**i**) and PVH (**j**). **i1**–**j1** Magnification of the frames indicated in the (**i**) and (**j**). Arrows indicated GAD2-positve neurons. Images are maximal projections of 100-μm z-stacks. **k** Horizontal and sagittal view of reconstruction of individual short-range and long-range neurons

From the low-resolution horizontal view of the brain, less dense yet significant projections of the infected GAD2 neurons are also found to project to the ventral posterior complex of the thalamus (VP) (Fig. 1a), a sub-region of the ventral group of the dorsal thalamus (VENT) responsible for somatosensory response. However, further experiments using a retrograde tracer, cholera toxin B (CTB) revealed that these VP-targeting projections were not originating from the NTS, but from the adjacent parasolitary nucleus (PAS) and DCN (including the gracile nucleus (Gr) and cuneate nucleus (CU)) (n = 3 mice) (Additional file 1: Fig. S2a, b), that were inadvertently infected due to spillover of the injected viruses. This is consistent with the observations that neurons in the Gr projects to the VP as shown in the Allen Brain Atlas [12], although these previously observed projections were not specifically labeled for GAD2 neurons.

Surprisingly, higher-resolution views further revealed long-range projection targets in various subcortical regions in the forebrain that were not shown in the Allen Brain Atlas. Clusters of axonal fibers were found in the bed nuclei of the stria terminalis (BST) (Fig. 1c) and the paraventricular hypothalamic nucleus (PVH) (Fig. 1d), both known for their roles in stress-response and emotional regulation [13]. Furthermore, formation of axonal arborizations in these areas as well as the SPV was confirmed by the branching and termination structures of the labeled fibers (Fig. 1b, b1-b2, c1-c2, d1-d2, Additional file 2: Video S1, Additional file 3: Video S2, Additional file 4: Video S3). Thus, inhibitory neurons in the NTS appear to also send out long-range projections to distant targets, in addition to local or short-range projections within the hindbrain as indicated in previous studies.

To confirm the GAD2-driven expression of Cre recombinase in the transgenic mice used in this experiment, we cross-bred them with a reporter line Ai14 (Rosa-CAG-LSL-tdTomato) and performed whole-brain imaging of tdTomato fluorescence (Additional file 1: Fig. S3). The brain-wide distribution pattern of fluorescent neurons was consistent with GAD2 expression shown in the Allen Brain Atlas [14], but with brighter fluorescence signals in corresponding areas, likely due to higher sensitivity of our 3D imaging approach.

To examine whether the long-range axonal projections to the BST and PVH originate from the NTS or the surrounding regions, we injected CTB into the BST and PVH (see Methods). CTB-positive neurons were found in the NTS but not in any adjacent regions including the DCN and PAS (n = 4 mice) (Additional file 1: Fig. S2c–f). To examine the cell-type of these long-range projections, we performed more CTB tracing from the BST and PVH in GAD2-Cre::Ai14 transgenic mice, in which GAD2 neurons expressed fluorescent protein tdTomato. Some of these CTB neurons in the NTS exhibited red fluorescence (13.0 ± 4.3% and 26.5 ± 7.7%, n = 20 and 14 slices from 3 mice for the BST and PVH respectively) (Fig. 1e–h), indicating that the NTS GAD2 neurons could indeed target these forebrain regions.

As a more direct test, we retrogradely labeled GAD2 innervations to the BST and PVH in GAD2-Cre mice with Cre-dependent rAAV2-retro expressing eGFP (Additional file 1: Fig. S4a, c) [15]. Similar to the CTB tracing results, sparse fluorescent somas were found in the NTS, but not the surrounding regions (Fig. 1i, j, Additional file 1: Fig. S4b, d), confirming that the GAD2 innervations to the BST and PVH did originate from the NTS.

Finally, we aimed to visualize the brain-wide morphology of these long-range projecting GAD2 neurons in the NTS using a sparse labeling strategy (see “Methods”) [16]. Different projection patterns were observed in 3 reconstructed neurons, each targeting one or more forebrain and midbrain regions including the BST, the paraventricular nucleus of the thalamus (PVT) and the periaqueductal grey (PAG) (Fig. 1k, Additional file 5: Video S4). In the same brain, we also traced 2 short-range projecting cells, targeting the SPV, PSV or the intermediate reticular nucleus (IRN) in the hindbrain for comparison (Fig. 1k, Additional file 5: Video S4).

In summary, we have discovered previously unknown long-range projections of the NTS GAD2 neurons that target forebrain areas including the BST and PVH, which are involved in diverse brain functions from energy balance to stress-coping and emotional regulation. Although only a small number of NTS GAD2 neurons are found to make such long-range projections, they may play important modulatory roles in these functions. It is noted that some cells might transiently express GAD2 during development but not act as inhibitory neurons later, although our use of adult animals for viral injection makes this scenario less likely. Systematic studies with sparsely labeling and reconstruction are expected to reveal a complete projectome of the NTS GAD2 neurons, and more insights regarding their role in visceral physiology, emotion and cognition.

## Supplementary Information

**Additional file 1: Fig. S1.** Serial coronal sections of the injection site in the NTS; **Fig. S2.** Distribution of CTB neurons in the NTS by retrograde tracing from the VP, BST and PVH; **Fig. S3.** Comparison of Cre-dependent tdTomato expressions in our GAD2-Cre mice to that in the Allen Brain Atlas; **Fig. S4.** Cre-dependent retrograde tracing from the BST and PVH using rAAV-retro.

**Additional file 2: Video S1.** Axonal arborizations in the SPV.

**Additional file 3: Video S2.** Axonal arborizations in the PVH.

**Additional file 4: Video S3.** Axonal arborizations in the BST.

**Additional file 5: Video S4.** Sparse labelling of NTS GAD2 neurons.

## Data Availability

All data presented are available upon reasonable request.

## Abbreviations

NTS: Nucleus of the solitary tract
GAD2: Glutamate decarboxylase 2
SPV: Spinal nucleus of the trigeminal
PSV: Principal sensory nucleus of the trigeminal
VP: Ventral posterior complex of the thalamus
BST: Bed nucleus of the stria terminalis
PVH: Paraventricular hypothalamic nucleus
CTB: Cholera toxin B
PHA-L: Phaseolus vulgaris‐leucoagglutinin
VISoR: Volumetric imaging with synchronized on-the-fly-scan and readout
PARN: Parvicellular reticular nucleus
DCN: Dorsal column nuclei
VENT: Ventral group of the dorsal thalamus
Gr: Gracile nucleus
CU: Cuneate nucleus
PAS: Parasolitary nucleus
PVT: Paraventricular nucleus of the thalamus
PAG: Periaqueductal grey
IRN: Intermediate reticular nucleus
MDRN: Medullary reticular nucleus
