# Supplementary material for: Long-range GABAergic projections from the nucleus of the solitary tract
Source: Mol Brain. 2021 Feb 19;14:38. doi: 10.1186/s13041-021-00751-4 (PMC7893933; doi:10.1186/s13041-021-00751-4)
Supplement: Supplementary file 1 — Additional file 1: Fig. S1. Serial coronal sections of the injection site in the NTS; Fig. S2. Distribution of CTB neurons in the NTS by retrograde tracing from the VP, BST and PVH; Fig. S3. Comparison of Cre-dependent tdTomato expressions in our GAD2-Cre mice to that in the Allen Brain Atlas; Fig. S4. Cre-dependent retrograde tracing from the BST and PVH using rAAV-retro. [file 13041_2021_751_MOESM1_ESM.docx]

Additional file 1

**Long -range GABAergic projections from the nucleus of the solitary tract**

Mei-Yu Shi, Lu-Feng Ding, Yu-Hong Guo, Yu-Xiao Cheng, Guo-Qiang Bi^*^ and Pak-Ming Lau^*^

*Correspondence: [plau@ustc.edu.cn](mailto:plau@ustc.edu.cn) or gqbi@ustc.edu.cn

**This file includes:**

Materials and Methods

Fig. S1

Fig. S2

Fig. S3

Fig. S4

**Materials and Methods**

**Animals**

All experimental procedures conducted in this study were in accordance with the international guidelines and protocols approved by the Institutional Animal Care and Use Committee of the University of Science and Technology of China. Female and male C57BL/6J, GAD2-IRES-Cre (Jackson Stock No: 010802) [1], Rosa‐CAG‐LSL‐tdTomato (Ai14; Jackson Stock No: 007914) [2] and Rosa-CAG-LSL-ZsGreen1 (Ai6; Jackson Stock No: 007906) [2] mice aging between 8 to 12 weeks were kept under a 12h light/dark cycle (6:00/18:00) in a temperature-controlled (22 °C) colony room with ad-libitum access to food and water.

**Surgery and virus injection**

Mice were anesthetized with pentobarbital sodium (100 mg/kg) and positioned in a stereotaxic apparatus (RWD Life Science). The skull above the NTS was carefully removed by dental drill. For dense labeling experiments, mice were bilaterally micro-injected with 500 nl of purified and concentrated AAV virus carrying vectors expressing Cre-dependent eGFP (AAV2/9-EF1α-DIO-eGFP, BrainVTA; virus titer: 5.5 × 10^12^ v.g/ml) [3] into the NTS (coordinated from bregma AP / ML / DV: -7.50 ~ 7.90 / ±0.15 / -5.25 mm) using a syringe micropump (Nanoliter 2000 Injector, WPI) at a rate of 30 nl/min, allowing expression of virus for at least 4 weeks before sacrifice.

For retrograde tracing with cholera toxin B (CTB) (Thermo Fisher Scientific), GAD2-Cre::Ai6, GAD2::Ai14 transgenic or C57BL/6J mice were micro-injected with 30-50 nl of CTB-AF488 or CTB-AF594 in the bed nuclei of the stria terminalis (BST) (AP / ML / DV: +0.25 / ±0.60 / -4.25 mm), paraventricular hypothalamic nucleus (PVH) (AP / ML / DV: -0.46 / ±0.14 / -5.25 mm) or ventral posterior complex of the thalamus (VP) (AP / ML / DV: -1.60 / ±1.53 / -3.60 mm) at a rate of 5 nl/min, allowing for 1 week of tracer expression before sacrifice.

For Cre-dependent retrograde tracing experiments, mice were bilaterally or unilateral micro-injected 250 nl of rAAV2-retro-EF1α-DIO-eGFP (BrainVTA; virus titer: 5.27 × 10^12^ v.g/ml) in the BST or PVH, allowing expression of virus for at least 3 weeks before sacrifice.

For sparse labeling experiments, a cocktail of 125 nl of AAV-EF1α-DIO-flp (BrainVTA; virus titer: 6.4 × 10^7^ v.g/ml) and 125 nl of AAV-EF1α-fDIO-EYFP-EYFP (BrainVTA; virus titer: 5.55 × 10^12^ v.g/ml) was micro-injected into the NTS, allowing expression of virus for at least 2 months before sacrifice.

**Tissue processing**

Mice were deeply anesthetized and transcardially perfused with 20 ml 0.1M phosphate buffer (PBS) at 37 °C and 4 °C, respectively, followed by perfusion with 4% paraformaldehyde (PFA) in 0.1M PBS. Extracted brains were kept in the hydrogel monomer solution (HMS) containing 4% PFA (Electron Microscopy Sciences), 4% acrylamide (Sigma), 0.05% bisacrylamide (Sigma), 0.0025% VA044 (Wako) in PBS (w/v) for post-fixation for 24 hours at 4 °C, and subsequently embedded in an equal volume mixture of 20% bovine albumin serum (Sigma) and HMS.

Brains were cut into 45-50 300-μm-thick consecutive slices in series for AAV tracing experiments and 80-μm-thick slices for CTB tracing experiments, both using a vibroslicer (Compresstome VF-300, Precisionary Instruments). The brain slices for AAV tracing experiment were transferred into clearing solution containing 4% Triton X-100 in PBS (w/v) with gentle shaking for 24 hours at 37 °C. Subsequently, the slices were rinsed in PBS for three times and kept in PBS until mounting.

**Microscopy**

Brain slices from each mouse were mounted on a glass slide with polymerized HMS. After three rinses, slides were immersed overnight in a refractive-index-matching solution with refractive index of 1.52. Fluorescent images were acquired by the VISoR system at a resolution of 1 × 1 × 2.5 μm^3^.

**Whole-brain image reconstruction**

Images were processed and reconstructed semi-automatically using custom developed algorithms and software as described previously [4]. The top and bottom surface planes of each reconstructed 3D slice were flattened by linear regression and interpolation. Adjacent slices were stitched together based on the edges and signature signals in the slices with rigid transformation followed by elastic deformation of local domains. For fiber tracing, axons were annotated semi-automatically using a custom software implementing the Virtual Finger technology [5] at 1-μm resolution to construct the whole axonal route of a single neuron. Image rendering was performed using Imaris (Oxford Instruments). For identification of the specific brain regions, images were manually registered to the Allen Mouse Brain Common Coordinate Framework [6].

**Data analysis**

Statistical analyses were performed using Origin 2018. Analysis results were expressed as mean ± SEM.

Reference

1. Taniguchi H, He M, Wu P, Kim S, Paik R, Sugino K, et al. A resource of Cre driver lines for genetic targeting of GABAergic neurons in cerebral cortex. Neuron. 2011;71:995-1013.

2. Madisen L, Zwingman TA, Sunkin SM, Oh SW, Zariwala HA, Gu H, et al. A robust and high-throughput Cre reporting and characterization system for the whole mouse brain. Nat Neurosci. 2010;13:133-140.

3. Su YT, Gu MY, Chu X, Feng X, Yu YQ. Whole-Brain Mapping of Direct Inputs to and Axonal Projections from GABAergic Neurons in the Parafacial Zone. Neuroscience Bulletin. 2018;34:485-496.

4. Wang H, Zhu QY, Ding LF, Shen Y, Yang CY, Xu F, et al. Scalable volumetric imaging for ultrahigh-speed brain mapping at synaptic resolution. Natl Sci Rev. 2019;6:982-992.

5. Peng HC, Tang JY, Xiao H, Bria A, Zhou JL, Butler V, et al. Virtual finger boosts three-dimensional imaging and microsurgery as well as terabyte volume image visualization and analysis. Nature Communications. 2014;5.

6. Wang QX, Ding SL, Li Y, Royall J, Feng D, Lesnar P, et al. The Allen Mouse Brain Common Coordinate Framework: A 3D Reference Atlas. Cell. 2020;181:936-+.


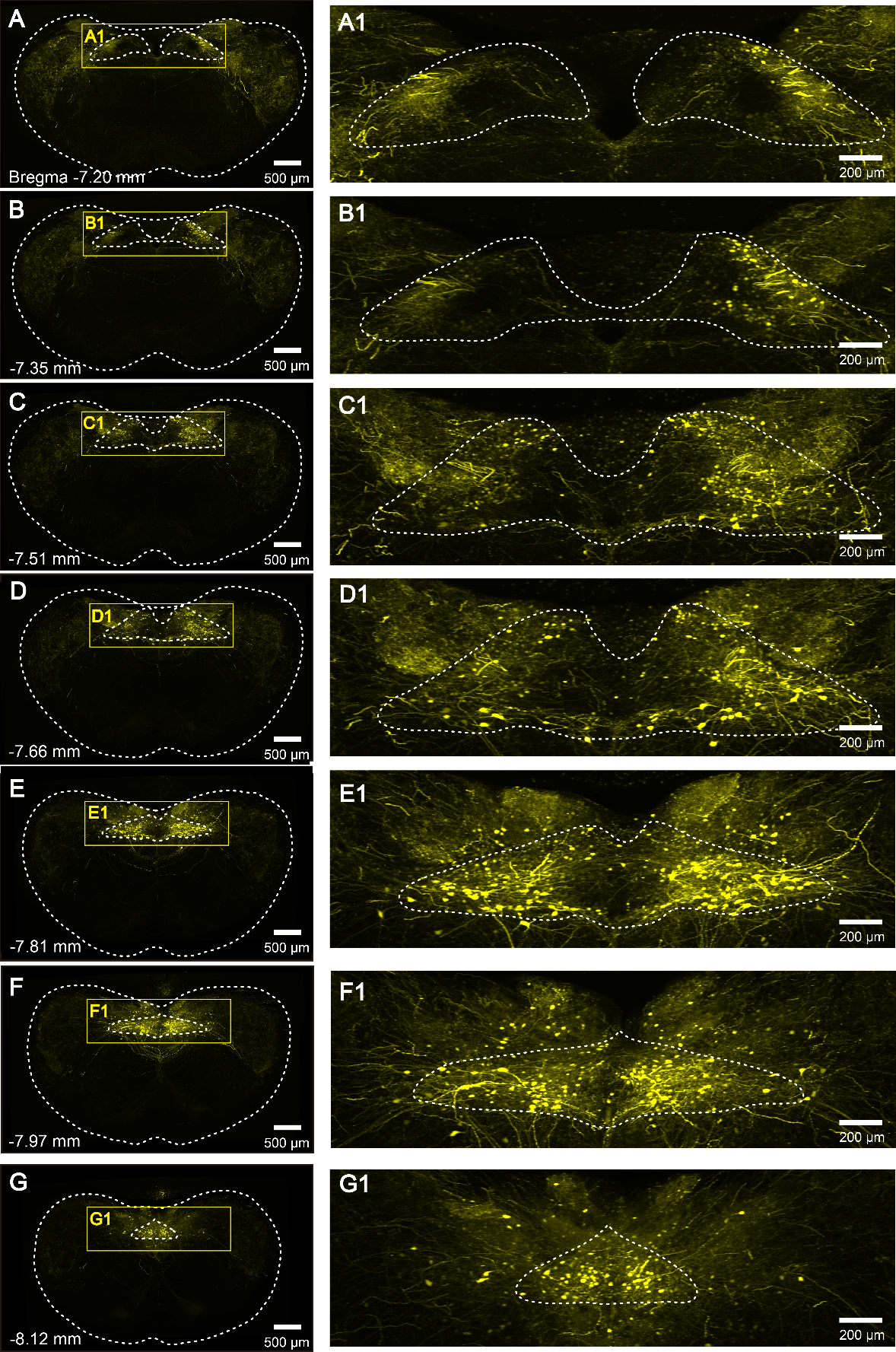


**Fig. S1. Serial coronal sections of the injection site in the NTS**

(**A-G**) Maximal projection views of representative coronal sections from the anterior to posterior NTS. (**A1-G1**) Magnified views of the virus injection site. Images are maximal projections of 256-μm z-stacks.


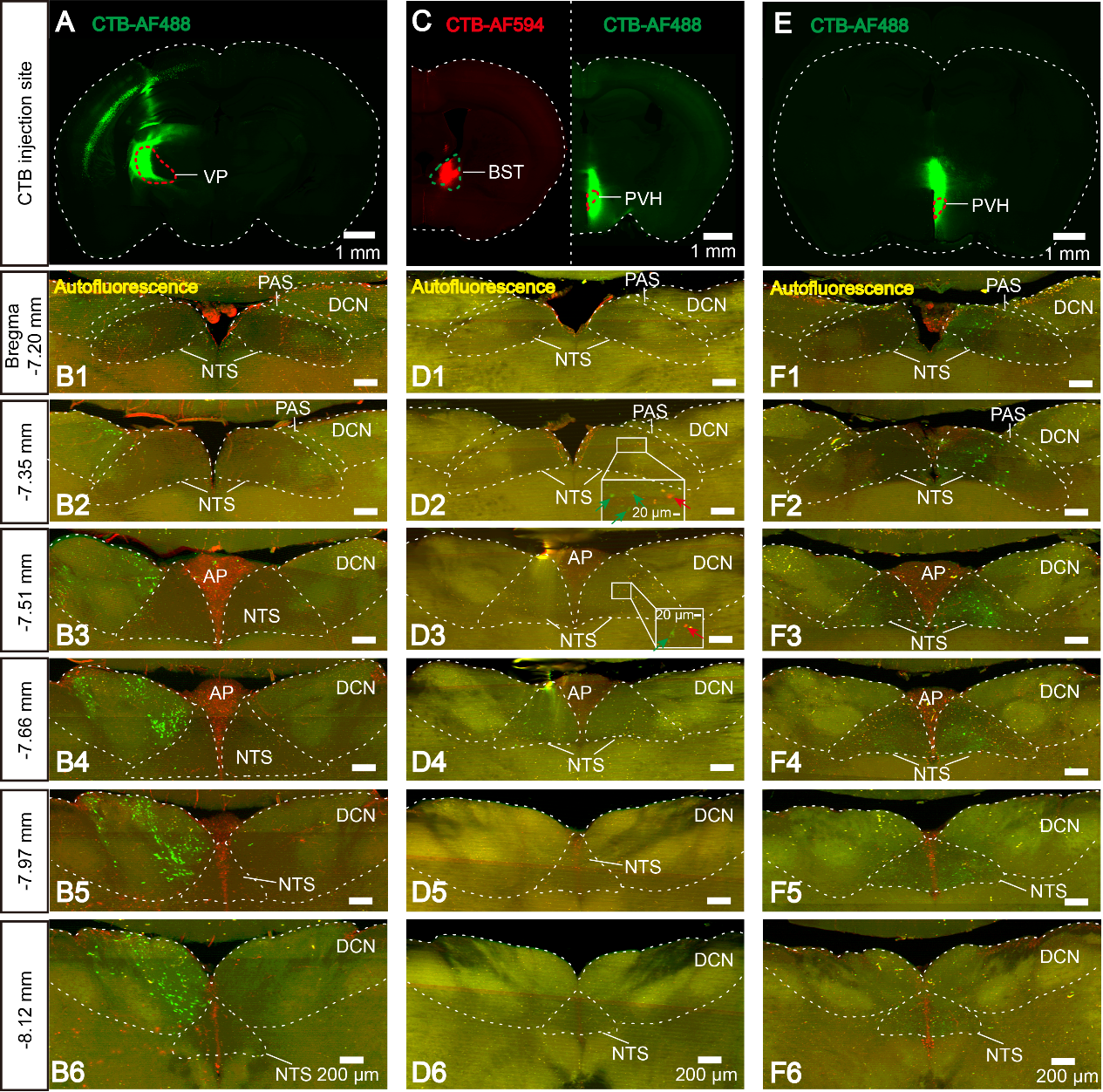


**Fig. S2. Distribution of CTB neurons in the NTS by retrograde tracing from the VP, BST and PVH.**

(**A, C, E**) Coronal sections indicating CTB injection sites in the VP (**A**), BST and PVH (**C**), and PVH (**E**), respectively. (**B, D, F**) Representative images showing expressions of CTB signals in the NTS, indicating retrograde tracing from the injected areas. The red and green arrows in **D2** and **D3** indicated BST- and PVH-projecting neurons respectively. Images are maximal projections of 100-μm z-stacks.


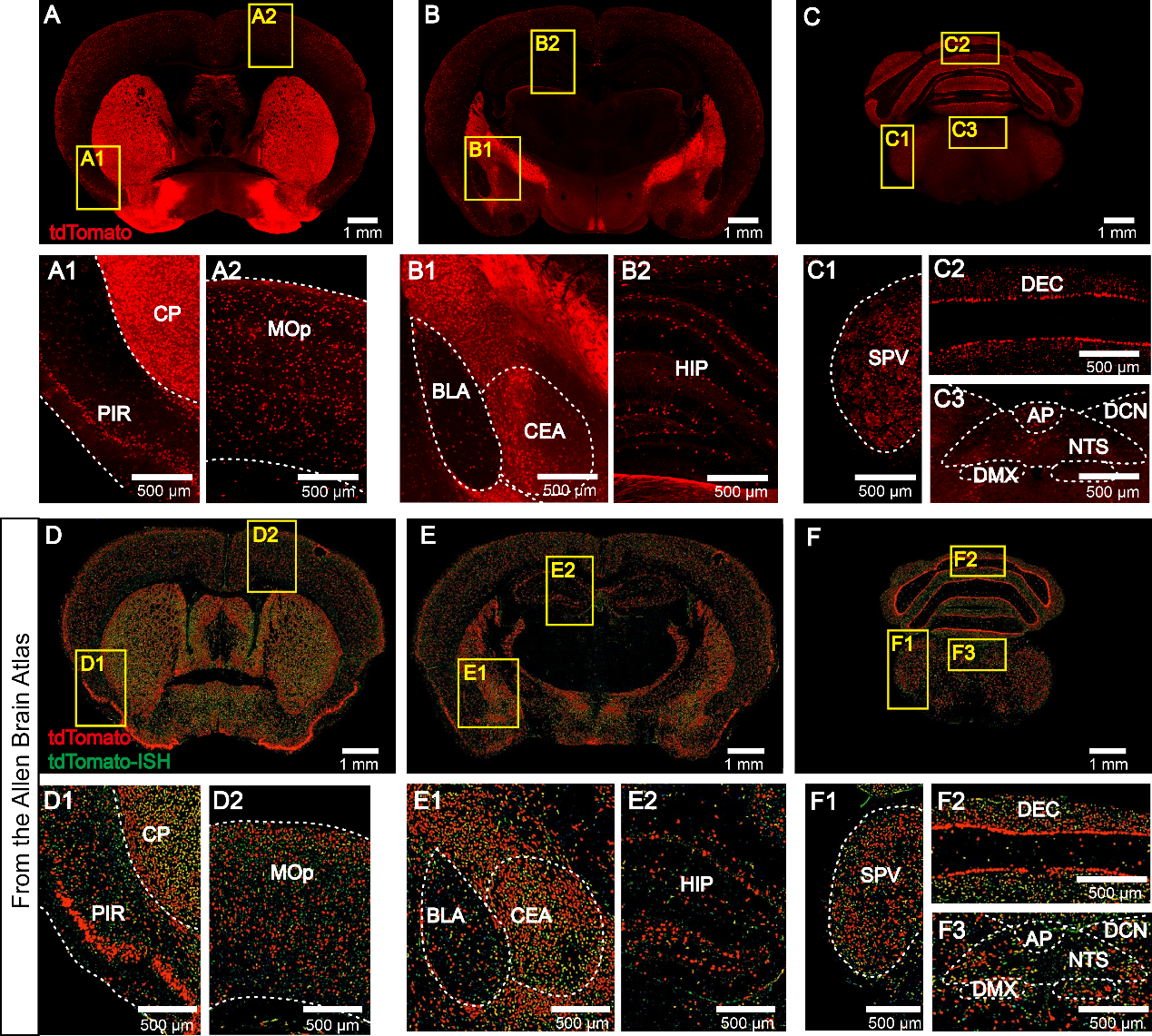


**Fig. S3. Comparison of Cre-dependent tdTomato expressions in our GAD2-Cre mice to that in the Allen Brain Atlas.**

(**A-C**) Coronal sections of tdTomato expressions in our GAD2-Cre::Ai14 mice. (**A1-A2**, **B1-B2**, **C1-C3**) Examples of tdTomato-labeled neurons in the piriform area (PIR), caudoputamen (CP), primary motor area (MOp), basolateral amygdala nucleus (BLA), central amygdala nucleus (CEA), hippocampal region (HIP), spinal nucleus of the trigeminal (SPV), declive (DEC) and NTS indicated in (**A-C**). (**D**-**F**) Coronal sections of GAD2-tdTomato expressions referenced to the Allen Brain Atlas. (**D1-D2**, **E1-E2**, **F1**-**F3**) Zoom-in views of tdTomato-labeled neurons in the brain regions indicated in (**D-F**).


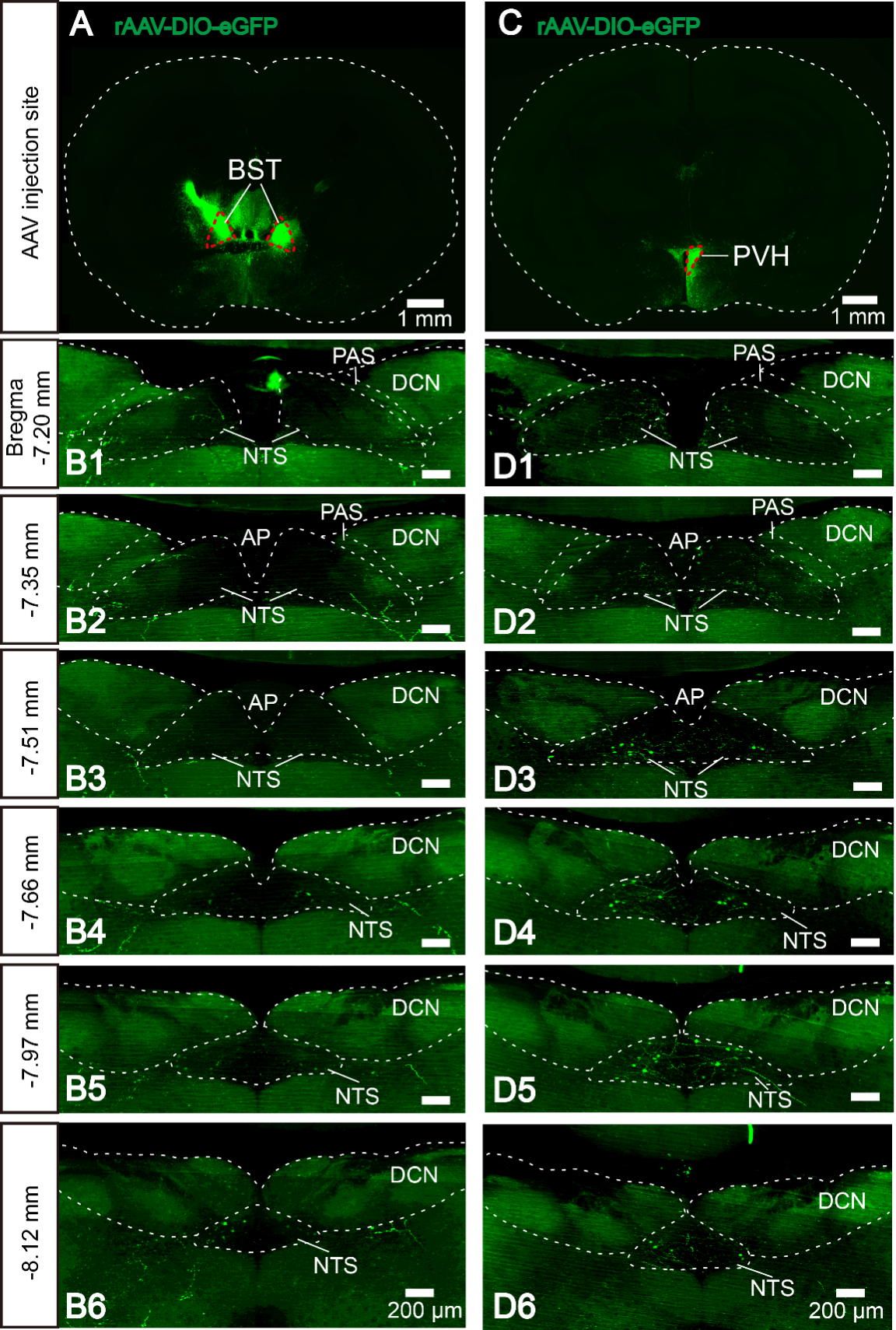


**Fig. S4.** **Cre-dependent retrograde tracing from the BST and PVH using rAAV-retro.**

(**A, C**) Coronal sections indicating rAAV2-retro-DIO-eGFP injection sites in the BST (**A**) and PVH (**C**). (**B, D**) Representative images showing fluorescent somas indicating BST- (**B1-B6**) and PVH- (**D1-D6**) projecting GAD2 neurons in the NTS. Images are maximal projections of 100-μm z-stacks.
